# A pairwise functional connectivity similarity measure method based on few-shot learning for early MCI detection

**DOI:** 10.3389/fnins.2022.1081788

**Published:** 2022-12-19

**Authors:** Xiangfei Zhang, Shayel Parvez Shams, Hang Yu, Zhengxia Wang, Qingchen Zhang

**Affiliations:** ^1^School of Cyberspace Security, Hainan University, Haikou, China; ^2^School of Computer Science and Technology, Shandong Technology and Business University, Yantai, China; ^3^School of Computer Science and Technology, Hainan University, Haikou, China

**Keywords:** Alzheimer's disease, few-shot learning, automatic diagnosis, smart medical, rs-fMRI

## Abstract

Alzheimer's disease is an irreversible neurological disease, therefore prompt diagnosis during its early stage, i.e., early mild cognitive impairment (MCI), is crucial for effective treatment. In this paper, we propose an automatic diagnosis method, a few-shot learning-based pairwise functional connectivity (FC) similarity measure method, to detect early MCI. We first employ a sliding window strategy to generate a dynamic functional connectivity network (FCN) using each subject's rs-fMRI data. Then, normal controls (NCs) and early MCI patients are distinguished by measuring the similarity between the dynamic FC series of corresponding brain regions of interest (ROIs) pairs in different subjects. However, previous studies have shown that FC patterns in different ROI-pairs contribute differently to disease classification. To enable the FCs of different ROI-pairs to make corresponding contributions to disease classification, we adopt a self-attention mechanism to weight the FC features. We evaluated the suggested strategy using rs-fMRI data obtained from the Alzheimer's Disease Neuroimaging Initiative (ADNI) database, and the results point to the viability of our approach for detecting MCI at an early stage.

## 1. Introduction

Recently, artificial intelligence has achieved exciting achievements in many fields (Zhou et al., 2020; Yu et al., 2021a,b; Wang S. et al., 2022). Benefiting from the progress of deep learning technology, computer-aided medical tools for neurological diseases have been developed and applied in a wide range of fields (Wang et al., 2017; Li et al., 2019; Zhao et al., 2022a). In terms of disease diagnosis, researchers have developed various auxiliary diagnostic tools (Li et al., 2017; Zhao et al., 2022b,c). For neurological diseases, however, although deep learning has become a promising method to diagnose neurological diseases, it is a serious challenge to learn potential features from real data using deep learning to diagnose neurological diseases, such as autism spectrum disorder and Alzheimer's disease (AD).

As the most common type of dementias in old age, AD is a neurological disorder that cannot be reversed. It's clinically characterized by gradual and progressive decline in memory as well as other cognitive functions, which increases the potential safety risks and even threatens life. Since AD cannot be cured to reverse its progression, early diagnosis is essential to delay AD progression and reduce potential risk at its initial prodromal stage, i.e., mild cognitive impairment (MCI), especially at early MCI (eMCI) stage (Kam et al., 2020). AD involves the disorder of brain structures and functions while the brain function of eMCI patients is expected to be greatly affected. Therefore, many eMCI studies focus on exploring its functional biomarkers for detecting eMCI (Chen et al., 2016; Kam et al., 2020; Wang et al., 2020).

Resting state functional magnetic resonance imaging (rs-fMRI), as an emerging noninvasive neuroimaging technology, utilizes blood oxygen level-dependent (BOLD) signals to measure spontaneous brain functional activities. Recently, many researchers have worked incredibly hard in detecting the potential diagnostic biomarkers of eMCI/AD based on rs-fMRI data. In the area of computer-aided diagnosis, machine learning has become one of the most important methods to effectively analyze rs-fMRI data for eMCI detection, which is beneficial to effectively comprehend the neurological basis of eMCI (Kam et al., 2020; Zhao et al., 2022d).

Since the human brain is particularly interconnected and well-organized system, the process of high-level cognition relying on the interactions among distributed brain regions of interest (ROIs). To quantify functional interactions between ROIs, the rs-fMRI data has been employed to the generate functional connectivity network (FCN) that the rich information exchange among ROIs (Wang et al., 2020). Functional connectivity (FC) analysis quantifies the temporal correlations of BOLD signals across spatially distant ROIs. According to an increase in the number of rs-fMRI reports that have concentrated on this research, many brain disorders including AD are typically accompanied with changes in the FC patterns. As a result, the analysis of FCN offers a chance to investigate the connections between ROIs, which is necessary to find promising biomarkers for AD diagnosis.

Both conventional machine learning methods such as support vector machine and deep learning methods such as recurrent neural network have been widely used in FC analysis in previous rs-fMRI researches (Kam et al., 2020; Wang et al., 2020; Zhao et al., 2022d). For instance, Wang et al. (2020) suggested using a spatial-temporal convolutional-recurrent neural network to extract FC features for AD progression prediction. Zhao et al. (2022d) constructed a multi-view higher-order FCN and extracted features from it for support vector machine classification. Most studies have found that FCs based on different ROI-pairs have inconsistent effects on model outcomes (Dai et al., 2014; Kam et al., 2020; Wang et al., 2020; Zhao et al., 2022d). Some FCs have made positive contributions to the model results to varying degrees, while some FCs played a negative role in the model outputs. In other words, among many FCN models, some FC features are unnecessary. Therefore, it is necessary to filter the features to eliminate redundant features or weaken the negative effects of irrelevant features on the model outputs. Generally, classical machine learning methods usually adopt statistical algorithms (e.g., *t*-test) to remove features weakly associated with diseases, while deep learning methods automatically learn multiple different disease-related feature abstractions from the input data.

**Figure 1:**
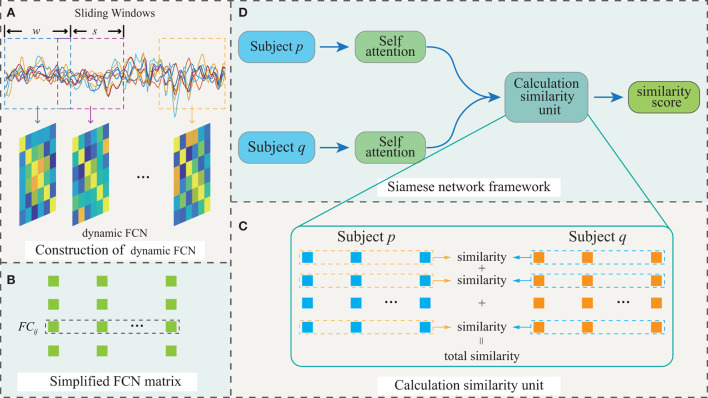
The framework of the proposed model. **(A)** Construction of dynamic FCN. **(B)** Simplified FCN matrix. **(C)** Calculation similarity unit. **(D)** Siamese network framework.

To this end, a novel pairwise FC similarity measure method based on few-shot learning and self-attention mechanism is proposed in this paper for eMCI detection. Our motivations are that (1) since different FC features may have different contributions to the model results, we employ the self-attention mechanism to weight FC features, so that FC feature can obtain an appropriate weight to properly affect the model outcomes; (2) Recently, metric learning is popular in classification tasks and has achieved satisfactory results (Jiang et al., 2021; Lai et al., 2021; Wang Y. et al., 2022). Therefore, we suggest that use metric learning to measure the similarity between pairwise FCs for eMCI vs. NC classification is feasible. Figure 1 illustrates the diagrammatic representation of the proposed framework, which consists of four parts, i.e., (1) construction of dynamic FCN, (2) simplified dynamic FCN, (3) calculation similarity unit, and (4) Siamese network framework. Specifically, we initially utilize the sliding-window approach (Wang et al., 2020; Zhao et al., 2022b,c) to construct a dynamic FCN, as recent progresses have consistently demonstrated that there is abundant diagnostic information on the inter-individual variability of FCN. Furthermore, due to the symmetric nature of the FCN matrix, only the lower off-diagonal triangular part of the FCN matrix are retained to avoid redundant computation. Further, the FC series are weighted by a self-attention mechanism, and the overall similarity between subjects is achieved by figuring out the similarity of its pairwise weighted FC time series. Finally, a popular metric learning/few-shot learning method, i.e., Siamese network, is trained to measure the similarity between subjects.

The major advantages of this study are three folds:

- *Firstly*, the self-attention mechanism is used to automatically assign weights to FC features.
- *Secondly*, the proposed model combines a self-attention mechanism and a few-shot learning strategy to facilitate feature learning and improves classification performance.
- *Thirdly*, systematic experiments are conducted on the large-scale ADNI dataset. The outcomes illustrate that the proposed method outperformed current cutting-edge classification techniques.

## 2. Materials and methods

The proposed model aims to detect whether subjects suffer from eMCI. To fulfill this purpose, we develop a pairwise FC similarity measure model, which consists of dynamic FCN, pairwise FC similarity measure module and Siamese network, as shown in Figure 1. Furthermore, experiments are carried out on the data from Alzheimer's Disease Neuroimaging Initiative (ADNI) database to verify the effectiveness of the proposed model.

### 2.1. Materials

1) Data acquisition: The ADNI database has been used to obtain the rs-fMRI data employed in this investigation. A total of 464 unprocessed rs-fMRI data from 95 MCI patients and 48 normal controls (NCs) were used.

This study's participants have been scanned with one or several appointments divided mostly by minimum of half a year, which is significant since using these 464 scans divided into 154 NC cases and 310 MCI cases (165 eMCI and 145 late MCI cases, correspondingly) as a result. Since we only used eMCI data and excluded late MCI data and ultimately used 319 scan cases (154 and 165 for NCs and eMCIs, correspondingly). The scanning period for each subject is 7 min, and the in-plane imaging resolution for each scan is 2.29–3.31 mm, slice thickness is 3.31 mm, TE (echo time) is 30 ms, and TR (repetition time) is 2.2–3.1 s. (resulting in 140 volumes). Table 1 provides a summary of the 319 subject's demographic data; values are expressed as the overall average variance. Male/Female: M/F.

**Table 1:** Demographic information of the studied subjects.

**Category**	**Scan #**	**Age (Years)**	**Gender (M/F)**
NC	154	75.36 ± 6.16	67/87
eMCI	165	72.03 ± 7.26	73/92

2) Data pre-processing: Using the FSL FEAT software, the rs-fMRI scans for each person under study were pre-processed according to a set manner. We mainly processed the remaining 137 volumes using a conventional pipeline, which included slice timing correction, head motion estimation, bandpass filtering, and regression of nuisance variables after initially discarding the initial three volumes during pre-processing for magnetization equilibrium (i.e., white matter, cerebrospinal fluid, and motion parameters). Subjects exhibiting head motions more than 2.0 mm in translation or 2.0 in rotation were disqualified. Along with that, we used T1-weighted MRI to accomplish structural skull stripping, and we aligned the skull-stripped fMRIs with the Montreal Neurological Institute (MNI) space. After that, a Gaussian kernel with a full-widthat-half-maximum (FWHM) of 6 mm was used to further spatially smooth the fMRI data. Because doing so might result in more artifacts, we didn't undertake scrubbing on data with frame-wise displacements >0.5 mm. The patients with a frame-wise displacement of more than 2.5 min (*FD*>0.5) were disqualified from further study. At last, using the AAL atlas, we retrieved the mean rs-fMRI time series (bandpass filtered from 0.015 to 0.15 Hz) from a collection of 116 pre-defined ROIs. Finally, the suggested method's input data consisted of time series of BOLD signals from all ROIs.

### 2.2. Dynamic FCN

Letting $\text{X}={\left({\text{x}}_{1},\ldots ,{\text{x}}_{N}\right)}^{T}\in {\mathbb{R}}^{N\times M}$ denote the rs-fMRI scanning series data of a subject, each vector ${\text{x}}_{n}\in {\mathbb{R}}^{M}\left(n=1,\cdots \;,N\right)$ contains the BOLD measurements of the *n*-th ROI at *M* sequential time points. To construct the dynamic FCN, the rs-fMRI scanning series is split into several overlapping subsegments by a sliding window with fixed length, and furthermore a short-term FCN is constructed for each subsegment. All the short-term FCNs compose the dynamic FCN for a subject. The workflow of constructing a dynamic FCN is shown in Figure 1A.

In detail, the rs-fMRI scanning series are partitioned into *K* = ⌊*M*−*w*⌋/*s*+1 overlapping subsegments, where *w* and *s* are the length and the step size of the sliding window, respectively. *k*-th (1 ≤ *k* ≤ *K*) window, the short-term FC between the *i*-th and the *j*-th ROIs is calculated by the Pearson's correlation coefficient:


(1) $$\begin{array}{l} FC_{ij}\left(k\right)=corr\left({\text{x}}_{i}\left(k\right),{\text{x}}_{j}\left(k\right)\right) \end{array}$$

where **x**_*i*_(*k*) denotes the sub-series in the *k*-th segment of *i*-th ROI. Thus, the shot-term FCN can be constructed as $\text{D}\left(k\right)=\left[FC_{ij}\left(k\right)\right]\in {\mathbb{R}}^{N\times N}$, and accordingly the dynamic FCN can be represented as **D** = [**D**(1), ⋯ , **D**(*k*), ⋯ , **D**(*K*)]∈ℝ^*K*×*N*×*N*^. For two specific ROIs, the FC series **FC**_*ij*_ = (*FC*_*ij*_(1), ⋯ , *FC*_*ij*_(*k*), ⋯ , *FC*_*ij*_(*K*)) reflects the dynamic FC between *i*-th and *j*-th ROIs.

Since each FCN matrix **D**(*k*) is symmetry, only the lower off-diagonal triangular part is used, i.e., $\text{D}\left(k\right)\in {\mathbb{R}}^{\frac{N\times \left(N-1\right)}{2}}$ (as shown in Figure 1B), so that redundant computation can be avoided to improve the training efficiency.

### 2.3. Pairwise FC similarity measure

The similarity of FC series between different subjects can serve as a feature to distinguish subject categories. Intuitively, the FC time series of ROI pairs between subjects from the same category should be more similar, while the FC series similarity between subjects from different categories should be lower. Therefore, a pairwise FC similarity measure between different subjects can be used to discriminate between NC and eMCI.

However, according to previous studies (Wang et al., 2020; Zhao et al., 2022d), FC between different ROI pairs has a distinct contribution degree to eMCI diagnosis. To this end, we assign a weight to each element of the FCN matrix with a self-attention mechanism as follows:


(2) $$\begin{array}{l} Query={\text{W}}_{Q}\text{D}\\ Key={\text{W}}_{K}\text{D}\\ Value={\text{W}}_{V}\text{D} \end{array}$$

where *Query* conveys the query, meaning that for each FC series is a enquiry to be asked, *Key* conveys the key which each query compares, *Value* conveys the value which extracts out the most important information from each FC series, and **W**_*Q*_, **W**_*K*_, **W**_*V*_ are learnable parameters. The weighted dynamic FCN matrix $\hat{\text{D}}$ is calculated using Equation (3):


(3) $$\begin{array}{l} \hat{\text{D}}=\mathrm{softmax}\left(\frac{{\text{Query Key}}^{T}}{\sqrt{d_{k}}}\right)\text{Value} \end{array}$$

where *d*_*k*_ denotes the dimension of *Key*. In our case, **D** and $\hat{\text{D}}$ are the same scale.

After the self-attention mechanism, $\hat{\text{D}}$ is used to calculate the pairwise FC series similarity of the subjects. As shown in Figure 1C, the similarity between the FC series of the corresponding ROIs between the two subjects is calculated and summed as the similarity between two subjects. Especially, the similarity is calculated by cosine similarity:


(4) $$\begin{array}{l} Cosine\left({\hat{FC}}_{ij}^{p},{\hat{FC}}_{ij}^{q}\right)=\frac{\sum_{k=1}^{K}{\hat{FC}}_{ij}^{p}\left(k\right)\cdot {\hat{FC}}_{ij}^{q}\left(k\right)}{\sqrt{\sum_{k=1}^{K}{\hat{FC}}_{ij}^{p}{\left(k\right)}^{2}}\sqrt{\sum_{k=1}^{K}{\hat{FC}}_{ij}^{q}{\left(k\right)}^{2}}} \end{array}$$

where ${\hat{FC}}_{ij}^{p}$ in $\hat{\text{D}}$ denotes the weighted FC series between *i*-th and *j*-th ROIs of *p*-th subject. Due to dynamic FCN is inherently time sensitive, that is, if the chronological order of the subnetworks of dynamic FCN changes, the dynamic FCN will also change (Chen et al., 2016; Zhao et al., 2022d). Therefore, ensuring the phase matching among the subnetworks of the subjects' dynamic FCNs is the key to achieve temporal consistency comparison. In Equation 4, since the element-wise computation for cosine similarity, $Cosine\left({\hat{FC}}_{ij}^{p},{\hat{FC}}_{ij}^{q}\right)$ is invariant to the order of elements in ${\hat{FC}}_{ij}^{p}$ and ${\hat{FC}}_{ij}^{q}$. Therefore, the phases of the subnetworks among subjects are matched, so that the meaningful comparisons can be made among subjects on temporal consistency.

### 2.4. Few-shot learning training strategy

1) Siamese network: In this study, we use the Siamese network as a concrete implementation of few-shot learning. The Siamese network consists of two subnetworks that share parameters, and in our model, the subnetworks consist of selfattention module. As illustrated in Figure 1D.

2) Dataset partitioning: The dataset used in this paper is divided into three parts, including training set, validation set and test set. The training set is used to learn the model parameters by optimizing the objective function. The training set and test set are further divided into support set and query set, respectively.

3) Objective function: In this work, the *ContrastiveLoss* (Hadsell et al., 2006) is employed as the objective function:


(5) $$\begin{array}{l} L=\left(1-y\right)s^{2}+y\max{\left(margin-s,0\right)}^{2} \end{array}$$

where *s* is the cosine similarity value, margin is the hyperparameters, and *y* is the label of a subject pair, if two subjects from same category, *y* = 1, otherwise, *y* = 0. When *y* = 1, a smaller loss denotes the similarity between subjects is bigger, while when *y* = 0, a smaller loss means that the similarity between subjects is smaller. Therefore, training the parameters aims to miminize the objective function. We use the backpropagation algorithm to train the parameters.

## 3. Results

### 3.1. Comparison methods

In the experiments, we compare the proposed method with the following three methods:

- **Support vector machine (SVM):** In this method, the average value of dynamic FC series is regarded as the features of ROI pairs. Therefore, for each subject, the size of the feature vector input into SVM is $\frac{N\times \left(N-1\right)}{2}$. In our case, *N* = 116.
- **Random forest (RF):** In this method, the input features are the same as those fed into the SVM.
- **Long short term memory (LSTM):** In this method, the input features are dynamic FCN matrices. Since LSTM is advantageous for processing sequence data, it is used to extract features from FC series. Finally, a *softmax* function is used for classification, and the objective function uses the *Crossentropy* *Loss*.

### 3.2. Experimental settings

In this study, the parameters of the sliding window are set as *w* = 30, *s* = 2, that is, the length of the sliding window is 30 time points or 90 s and the size of each transition is 2 time points or 6 s. In this paper, we report the 2-way (i.e., NC vs. eMCI) 5-shot evaluation results.

To verify the effectiveness of the proposed method and the comparison methods, four criteria, i.e., classification accuracy (ACC), specificity (SPE), positive predictive value (PPV) and negative predictive value (NPV), are used.


(6) $$\begin{array}{l} ACC=\frac{TP+TN}{TP+TN+FP+FN} \end{array}$$

(7) $$\begin{array}{l} SPE=\frac{TN}{TN+FP} \end{array}$$

(8) $$\begin{array}{l} PPV=\frac{TP}{TP+FP} \end{array}$$

(9) $$\begin{array}{l} NPV=\frac{TN}{TN+FN} \end{array}$$

where TP, TN, FP, and FN represent True Positive, True Negative, False Positive, and False Negative, respectively.

### 3.3. Classification performance

The classification results of 5-shot in NC vs. eMCI are shown in Table 2. As Table 2 demonstrates that our method achieves 78% ACC value, 80% SPE value and so on. Obviously, the proposed method generally achieved better performance than the comparison methods. For example, in terms of ACC, the proposed method outperforms the best comparison method (i.e., LSTM) by 6.33%; and in terms of PPV value, the proposed method achieved the improvement of 11.60%, compared to the most effective comparison method (i.e., LSTM). These results show that explicit modeling of the pair-wised FC similarity measure in rs-fMRI time series comparing various subjects to capture eMCI-related ROIs in brain FC is helpful in predicting the AD progression.

**Table 2:** Performances of the proposed method and the comparison methods.

**Method**	**ACC (%)**	**SPE (%)**	**PPV (%)**	**NPV (%)**
RF	66.00	56.00	63.33	70.00
SVM	65.00	50.00	61.54	71.43
LSTM	71.67	60.00	67.57	78.26
Ours	78.00	80.00	79.17	76.92

## 4. Discussion

### 4.1. Pairwised FC similarity

**Figure 2:**
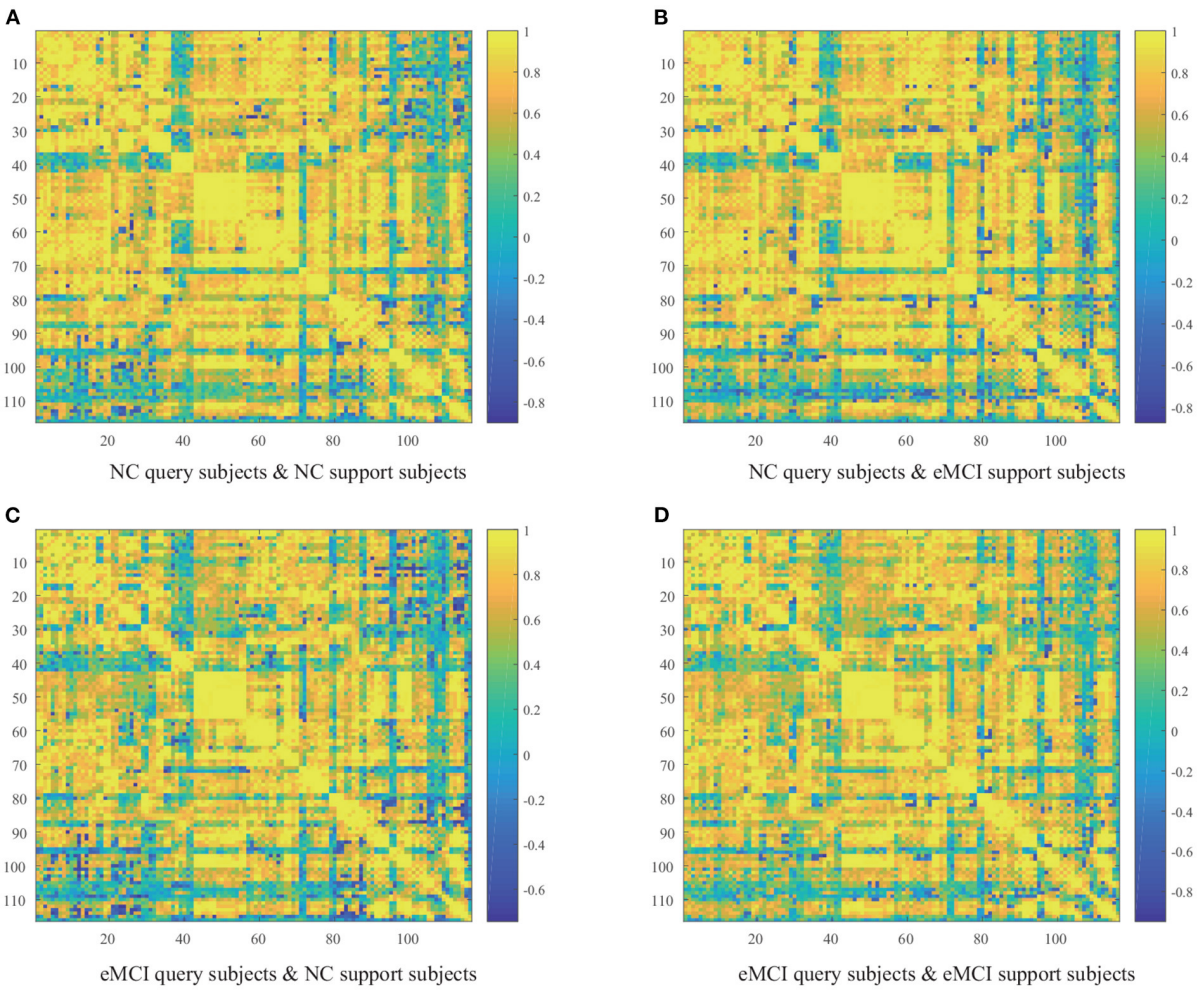
Average similarity matrix between query subjects and support subjects. **(A)** NC query subjects and NC support subjects. **(B)** NC query subjects and eMCI support subjects. **(C)** eMCI query subjects and NC support subjects. **(D)** eMCI query subjects and eMCI support subjects.

To discover abnormal patterns of dynamic FC in eMCI patients, Figure 2 presents four groups (i.e., NC query set and NC support set, NC query set and eMCI support set, eMCI query set and NC support set, and eMCI query set and eMCI support set) average similarity.

From Figure 2, we can draw several fascinating observations. *First*, the cosine similarity values of the query set and the support set are mostly positive, probably because the dynamic FC of eMCI patients did not change significantly. *Second*, more negative cosine similarities appear when the query set and support set are from different categories, which is in line with our expectations. When more negative similarity values appear, it means that their sum will be smaller, which is in line with our goal of less similarity between subjects from different classes. *Finally*, the four heatmaps show overall similarities and local differences. This is because the abnormal FC pattern in eMCI patients occurs between a few ROIs, not the whole brain FC pattern changes.

### 4.2. Discriminative pairwised ROIs

**Figure 3:**
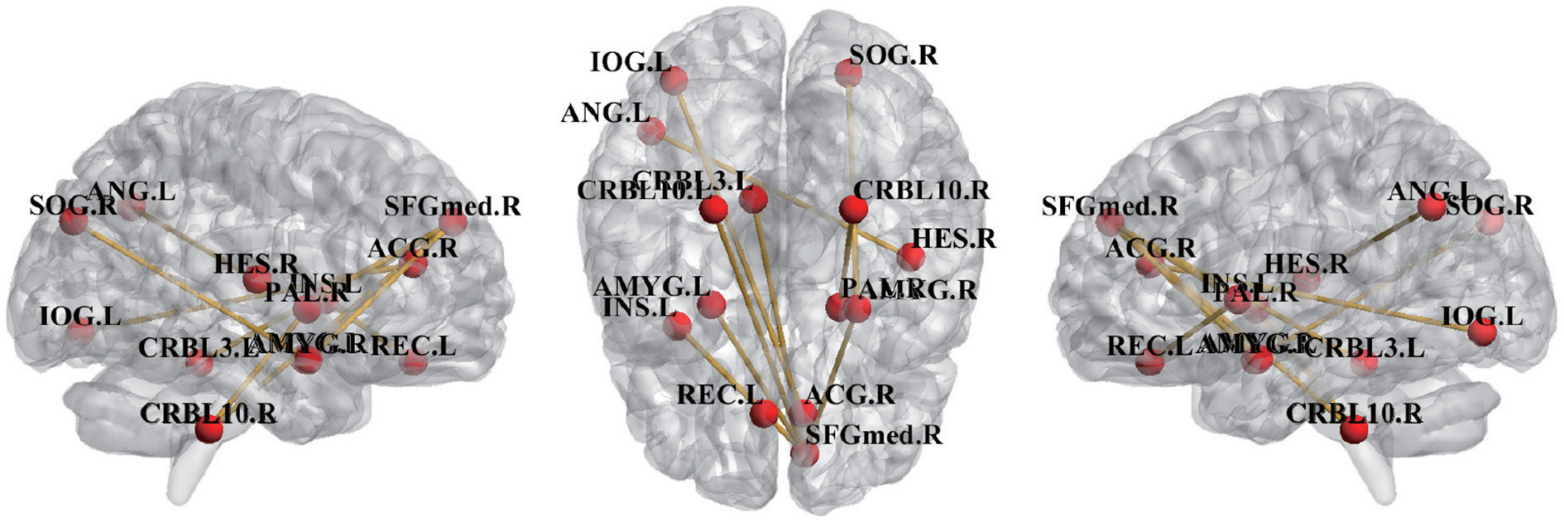
Top 10 pairwised ROIs identified by the proposed method.

To find the most discriminative ROI pairs, we compared the difference in mean similarity values between different queries on the same support set. Specifically, we compared the differences in similarity between two different query sets and support sets when NC subjects and eMCI subjects were used as support sets, respectively, with the aim of discovering the FCs that were different between eMCI query subjects and NC query subjects. Under the same support set, the ROI pairs with greater similarity difference are considered as the most discriminative pairwised ROIs, and the top ten most discriminative ROI pairs are shown in the Figure 3.

From Figure 3, we can see that the discriminative FCs and brain ROIs are extended out over both hemispheres which is a common distribution in previous studies (Wee et al., 2016; Zhao et al., 2022d), demonstrating the pattern of functional impairments that is dispersed throughout the whole brains of eMCI patients. Several brain ROIs, *right superior frontal gyrus* (SFGmed.R), *left rectus gyrus* (REC.L), and *right anterior cingulate gyrus* (ACG.R), belong to the default mode network (DMN), according to previous studies (Xia et al., 2013; Suk et al., 2016). DMN, which are selected as the ROI in eMCI identification that are the most discriminative and show a strong connection with the ROI in the higher-level cognitive functional networks (Seeley et al., 2007). Several brain ROIs, CRBL3.L, CRBL10.L, and CRBL10.R, belong to cerebellum regions. Researches has shown that patients with MCI experienced structural degenerative changes in some cerebellum regions (Wee et al., 2016) and disruption of cerebellum functions (Wee et al., 2016). The functions of *left angular gyri* (ANG.L) are maintaining attention, manipulating controls, working memory, making decisions, among many of others (Michael, 2005; Etienne and Christopher, 2007), and this region has been closely related to eMCI in previous reports (Liang et al., 2012; Zhou et al., 2013). The brain ROIs identified by uor method are consistent with previous reports, indicating the effectiveness of our proposed method.

## 5. Conclusions

In summary, a pairwise FC similarity measure method based on few-shot learning is proposed in this work for eMCI detection. The unique property of this method is combination of a self-attention mechanism to automatically learn the weights of FC features, which is beneficial for disease classification using whole-brain ROIs. This idea offers an encouraging solution to identify and categorize brain FCNs, and can also be expanded to FC-based diagnostic research in other brain diseases.

In this research, we focus on the automatic identification of eMCI using only rs-fMRI data. Indeed, it is possible to diagnose eMCI with the help of different imaging modalities, such as structural MRI and fluorodeoxyglucose PET. It is also intriguing to use multimodal Data for eMCI detection, which will be the focus of our upcoming work. In addition, the dataset is still insufficient even though we use all rs-fMRI scans from all individuals of ADNI database. In further research, we'll assess the proposed methods using a large-scale dataset with more brain neurological disease like autism spectrum disorder.

## Data availability statement

Publicly available datasets were analyzed in this study. This data can be found at: https://adni.loni.usc.edu/.

## Author contributions

XZ: formal analysis, investigation, methodology, and writing-original draft. SS and HY: review and editing. ZW: data curation and funding acquisition. QZ: funding acquisition, project administration, and conceptualization. All authors contributed to the article and approved the submitted version.
